# Predicting ALS informant distress from cognitive and behavioural change in people with ALS

**DOI:** 10.1007/s00415-024-12847-7

**Published:** 2025-01-15

**Authors:** Lyndsay Didcote, Silia Vitoratou, Ammar Al-Chalabi, Laura H. Goldstein

**Affiliations:** 1 Department of Psychology, Institute of Psychiatry, Psychology and Neuroscience, King’s College London, London, UK; 2 Department of Biostatistics and Health Informatics, Institute of Psychiatry, Psychology and Neuroscience, King’s College London, London, UK; 3 Department of Basic and Clinical Neuroscience, Maurice Wohl Clinical Neuroscience Institute, King’s College London, London, UK; 4 Department of Neurology, King’s College Hospital NHS Foundation Trust, London, UK

**Keywords:** Amyotrophic lateral sclerosis, Informant, Distress, Cognition, Behaviour

## Abstract

**Background:**

The cognitive and behavioural changes that occur in around 50% of people with amyotrophic lateral sclerosis (ALS) may significantly affect people around them, contributing to heightened burden, anxiety, and depression. Despite existing evidence linking behavioural impairment to caregiver distress, the role of cognitive impairment remains less clear, with mixed findings on its impact.

**Methods:**

This study assessed the influence of cognitive and behavioural impairments in people with ALS on the distress of their nominated informants. The data were collected face-to-face and remotely due to the COVID-19 pandemic. The cognitive and behavioural impairments were measured using established screening tools. Informants’ distress was evaluated through composite measures of burden, anxiety, and depression. Regression analyses were employed to determine the predictive value of cognitive and behavioural impairment on informant distress.

**Results:**

A total of 98 ALS patients and 84 informants participated. Behavioural impairment predicted informant distress across various tools. In contrast, cognitive impairment was a less consistent predictor of informant distress across screening measures and did not significantly interact with behavioural impairment in predicting distress. Administration mode did not affect predictive relationships.

**Conclusions:**

Behavioural impairment in ALS significantly predicts informant distress, with varying predictive power across different screening tools. Cognitive impairment also affects informant distress, but its impact is less substantial compared to behavioural factors. The interaction between cognitive and behavioural impairments did not significantly predict informant distress.

**Supplementary Information:**

The online version contains supplementary material available at 10.1007/s00415-024-12847-7.

## Introduction

The life-limiting, progressive neurodegenerative condition, amyotrophic lateral sclerosis (ALS), often referred synonymously as motor neuron disease (MND) involves the progressive weakness of limb, bulbar, and core muscles which leads to challenges with mobility, speaking, swallowing, chewing, and respiration [1, 2]. Around 50% of those diagnosed with ALS exhibit changes to cognition or behaviour, with approximately 15% of these presenting with ALS-frontotemporal dementia (ALS-FTD) [3, 4]. Cognitive symptoms include the impairment of executive function, social cognition, verbal fluency, and language function [5]. Behavioural symptoms include but are not limited to apathy, loss of sympathy or empathy, perseverative, stereotypic, or compulsive/ritualistic behaviour, hyperorality and dietary changes, loss of insight, and occasionally psychotic symptoms [5, 6]. Some people, however, do not experience any cognitive or behavioural symptoms.

There is evidence that caregivers of people with ALS experience raised levels of caregiver burden, anxiety, and depression [7–11]. The impact of behavioural impairment in ALS on caregiver burden is well documented [9, 12–18]. A systematic review examined factors influencing caregiver burden in informal caregivers of people with ALS and found high quality evidence for the association between caregiver burden and ALS behavioural impairment [9]. Regression analysis with a sample of 35 ALS spouse-caregivers demonstrated that ALS behavioural problems were predictive of caregiver burden [17]. An additional study, using a sample of 84 ALS-caregiver couples, found a correlation between caregiver burden and ALS behavioural impairment scores [16]. Elsewhere, caregivers with higher levels of burden reported higher levels of apathy in the respective people with ALS; only behavioural symptoms of apathy (and not cognitive, emotional and non-emotional symptoms of apathy) in the people with ALS explained a significant amount of variance in caregiver burden [18].

There is some limited evidence that cognitive impairment in the person with ALS also influences caregiver burden [12–15]. In a study primarily focusing on behavioural impairment and caregiver burden, researchers found that caregiver burden was strongly correlated with memory and orientation, and everyday skills [12] in the person with ALS. In a different sample of 43 ALS-caregiver pairs, an increase in caregiver burden over approximately 7 months was predicted by increases in cognitive and behavioural impairment over the same period [13]. A further study of 33 ALS-caregiver pairs found that higher burden in caregivers was predicted by executive dysfunction, as well as apathy and disinhibition [14] in the person with ALS. Similarly, another study of 70 ALS-caregiver couples reported that cognitive and behavioural symptoms, specifically executive dysfunction and apathy, in the person with ALS were related to caregiver burden, [15]. However, other studies have found that the presence of cognitive impairment in someone with ALS and caregiver burden are not related [9, 16, 17].

A link has been found between caregiver depression and the behavioural change that may occur in ALS, in a study of 70 ALS-caregiver pairs [15]. However, not all findings are consistent with this as a later study did not find that behavioural impairment in the person with ALSs predicted caregiver depression in a final regression model once other variables, such as physical function, had been taken into account [17]. The same study of 35 ALS spouse–caregiver couples also provided some limited evidence that behavioural change in people with ALS predicts caregiver anxiety [17]. That study also investigated cognitive impairment in ALS as a predictor of anxiety and depression in caregivers but did not find this to be the case [17].

This study focuses on people with ALS and their nominated informants, i.e., someone who knows them well such as a caregiver, relative, or friend. It aims to assess whether cognitive and behavioural change in people with ALS, or an interaction between these, are predictive of informant distress operationalised as a composite variable of burden, anxiety and depression. By investigating a possible interaction between cognitive and behavioural involvement in ALS on caregiver distress, it will be possible to explore whether the presence of both types of change have a synergistic effect when considering distress in the people around them.

## Methods

The data were collected for this study in two formats. Initially, the data were collected face-to-face, in person between 29 January 2019 and 10 March 2020 but the coronavirus pandemic prevented further face-to-face data collection. Therefore, subsequent data were collected remotely between 23 September 2020 and 17 June 2021.

### Recruitment and consent

In face-to-face and remote conditions, the participants with ALS were recruited from four multidisciplinary National Health Service (NHS) MND clinics, namely at King’s College Hospital NHS Foundation Trust, St George’s University Hospitals NHS Foundation Trust, Brighton and Sussex University Hospitals NHS Trust, and University Hospitals Dorset NHS Foundation Trust. In these NHS sites, care is free at the point of delivery. In the remote condition, people with ALS were also recruited through online advertising by the MND Association, social media, and classified advertisement websites. These ALS participants then nominated someone who knew them well such as a caregiver, relative, or friend to be an informant participant.

Participants with ALS were eligible if they had a diagnosis consistent with El Escorial criteria, could provide informed consent, and were 18 years or older. For those in the remote condition, additional requirements included having access to a device such as a desktop PC, laptop, or tablet (with a minimum 10-inch diagonal screen) equipped with a webcam, a microphone, speakers, and a wi-fi connection capable of supporting videoconferencing. The exclusion criteria included having diagnoses of progressive muscular atrophy or primary lateral sclerosis; being over 75 years old; non-native English speakers; a history of head injury, stroke, or other neurodegenerative diseases (except ALS with or without FTD); daytime use of non-invasive ventilation; a score higher than 10 on the Epworth Sleepiness Scale (ESS) [19], which indicates excessive daytime sleepiness; or ongoing treatment for another life-threatening illness.

Informant participants were included if they were at least 18 years old and had the ability to give consent. In the remote condition, participants needed access to the internet. The informants were excluded if they were unable to complete the measures independently.

### Data collection

#### Cognitive assessment of people with ALS

The focus of this research is on informant distress; analysis of data from the cognitive screening tools is published elsewhere [20].

In the face-to-face condition, the people with ALS were visited at their homes for data collection. In the remote condition, people with ALS were emailed a link to a videoconferencing call with the researcher at a pre-specified date and time. Videoconferencing calls were conducted using Skype.

Three cognitive screening tests were administered to the person with ALS in a pseudorandomised order. The cognitive screens included the cognitive component of the Edinburgh Cognitive and Behavioural ALS Screen (ECASc) [21], the cognitive component of the ALS Cognitive Behavioural Screen (ALS-CBSc) [22] and the Mini-Addenbrooke’s Cognitive Examination (Mini-ACE) [23], which was originally devised for detecting cognitive impairment in people with dementia although it has been suggested that it could be applied to people with ALS (see Table 1 in Online Resource 1). See Online Resource 1 for a description of how the cognitive screens were adapted for remote administration.

#### Informant completed measures

Informants in the face-to-face administration condition mostly took part at the same time as the person with ALS at their home, where they completed the following behavioural measures: the behavioural component of the Edinburgh Cognitive and Behavioural ALS Screen (ECASb) [21]; the behavioural component of the ALS Cognitive Behavioural Screen (ALS-CBSb) [22], the ALS-FTD Questionnaire (ALS-FTD-Q) [24], the Beaumont Behavioural Inventory (BBI) [25], and the Motor Neuron Disease Behavioural Instrument (MiND-B) [26] (see Table 2 in Online Resource 1). Informants also completed the following distress measures: the Generalised Anxiety Disorder Assessment 7-item scale (GAD-7 [27]); the Patient Health Questionnaire-9 depression scale (PHQ-9 [28]); the Zarit Burden Interview—short version (ZBI [29]; see Table 3 in Online Resource 1. Some informants who were unable to be present at that time completed the measures independently and returned them via post within 2 weeks of the person with ALS taking part.

Informants in the remote administration condition were given a link to a Qualtrics [30] questionnaire comprising the five measures of behavioural change and the three measures of informant distress. The informants were required to complete the measures within 2 weeks of the person with ALS participating in the study. The ECASb was originally intended to be administered through interviews conducted by clinicians, rather than as a self-completed questionnaire. For use in the remote condition, the ECASb was adapted for self-administration; however, the only modifications involved adjusting the instructions for the online format without altering the content of the questionnaire. This adaptation was authorised by the author of the scale.

The behavioural and distress measures were administered in a pseudorandomised manner (across all measures).

### Statistical analysis

Spearman’s correlations were carried out to assess the relationships between informant anxiety, depression and burden, due to the skewness of the data.

As scores on the PHQ-9, GAD-7, and ZBI were intercorrelated (see Table 4 in Online Resource 1), a composite measure “informant distress” was constructed to reduce the number of calculated regression models and minimise multiple comparisons.

The composite variable was calculated similarly to the global outcome measure of informants’ overall psychological status in Goldstein et al. [31]. For each measure, z-scores were calculated and the average of the three z scores formed the composite measure “informant distress”.

$$\frac{1}{3}\left[ {\frac{{{\text{observed}} {\text{GAD - 7}} - {\text{mean}}\; \left( {\text{GAD - 7}} \right)}}{{S.D. \left( {\text{GAD - 7}} \right)}} + \frac{{{\text{observed}} {\text{PHQ - 9}} - {\text{mean}}\; \left( {\text{PHQ - 9}} \right)}}{{S.D. \left( {\text{PHQ - 9}} \right)}} + \frac{{{\text{observed ZBI}} - {\text{mean}}\; \left( {{\text{ZBI}}} \right)}}{{S.D. \left( {{\text{ZBI}}} \right)}}} \right]$$

Multiple linear regression models that assessed the ability of cognitive and behavioural screening tools to predict informant distress were built using a forward stepwise method. The demographic variables that were entered into the models were selected if there was evidence in the literature that they might affect caregiver distress (caregivers of people with ALS and people with other conditions). Therefore, the revised ALS Functional Rating Scale score (ALSFRS-R) [17, 32], age of person with ALS [17], age of informant [33], number of dependents other than the person with ALS [31], sex of informant [15, 33, 34], informant employment status (employed/other) [33], relationship type (spouse/other) [34], and hours per week of caregiving [33, 35] were entered into all regression models individually during step one (univariate regression models). Variables that were significantly different between face-to-face and remote conditions (such as the proportion of people self-identifying as a caregiver rather than an informant) were also entered into linear regression models at step one.

In step two, significant predictor variables from step one were entered into the model together, starting with the variable with the lowest p-value of the largest effect size. If variables were no longer significant predictors in the resulting model, they were removed during step three. The final regression models, therefore, contained these predictor variables: a behavioural or cognitive screen score; other variables that significantly predicted informant distress; and administration mode (whether measures were administered remotely or face-to-face). The administration mode was entered into regression models as a predictor variable, whether significant or not [20], to control for this difference in data collection methods.

To determine whether ALS with cognitive impairment and ALS with behavioural impairment interact to predict informant distress, a multiple regression was conducted with the behavioural screening tool which was the predictor for which the univariate regression model had the highest coefficient of determination (R^2^) value, the cognitive screening tool which was the predictor for which the univariate regression model had the highest R^2^ value, and the interaction between the two screens as predictor variables, alongside the covariate “administration mode” (measures administered face-to-face or remotely).

Power was above 0.95 for 7/9 regression analyses according to a posteriori power analysis [36]. All analysis was conducted using SPSS 27 [37]; results were considered significant if p values < 0.05.

## Results

### Sample size

Ninety-eight people with ALS participated in the study, with 41 taking part face-to-face and 57 taking part online (see Table 1). A total of 84 informants participated in this research, 35 of whom did so face-to-face and 49 of whom did so remotely; see Figs. 1 and 2 in Online Resource 1). While the majority of informants were spouses, 12 were classified as being “other family member” and 9 were “friends”. The missing cognitive data were due to factors such as participants deciding not to participate, lack of engagement, and unsafe testing environments. Missing informant data were a result of informants deciding not to complete all of the measures.

**Table 1:** Available samples for each screening tool and measures of psychological and psychosocial distress in informant

	F2F^a^ (n)	REM^b^ (n)	Combined (n)
ECASc^c^	35	49	84
ALS-CBSc^d^	35	49	84
Mini-ACE^e^	35	49	84
ECASb^f^	35	48	83
ALS-CBSb^g^	35	49	84
BBI^h^	32	49	81
MiND-B^i^	35	48	83
ALS-FTD-Q^j^	35	48	83
PHQ-9^k^	35	48	83
GAD-7^l^	34	48	82
ZBI^m^	35	48	83

^a^Face-to-face

^b^Remote

^c^Cognitive component of the Edinburgh Cognitive and Behavioural ALS Screen

^d^Cognitive component of the ALS Cognitive Behavioural Screen

^e^Mini-Addenbrooke’s Cognitive Examination

^f^Behavioural component of the Edinburgh Cognitive and Behavioural ALS Screen

^g^Behavioural component of the ALS Cognitive Behavioural Screen

^h^Beaumont Behavioural Inventory

^i^Motor Neuron Disease Behavioural Instrument

^j^ALS-FTD Questionnaire

^k^Patient Health Questionnaire-9

^l^Generalized Anxiety Disorder Assessment

^m^Zarit Burden Interview—short version

### Behavioural measures

Estimated IQ (determined with the Test of Premorbid Functioning [38]) of the people with ALS and whether or not participants identified as a caregiver (i.e., they reported new caregiving duties following the onset of ALS symptoms) were entered into regression models during step one as these variables were significantly different between participants in the face-to-face and remote conditions (see Table 2).

**Table 2:** Differences in demographic variables in the ALS and informant samples between face-to-face and remote testing conditions

		*n* (%)		*χ*^*2*^ (df)
		REM^a^	F2F^b^	
Sex of person with ALS^c^	Female	20 (35.1)	22 (53.7)	3.358 (1)
	Male	37 (64.9)	19 (46.3)	
Sex of informant	Female	33 (67.4)	24 (68.6)	0.014 (1)
	Male	16 (32.7)	11 (31.4)	
ALS: method of communication	Format prescribed by measure	25 (43.9)	17 (42.5)	1.723 (2)
	Written only	7 (12.3)	2 (5.0)	
	Spoken only	25 (43.9)	21 (52.5)	
ALS: symptom onset region	Bulbar	12 (21.1)	7 (17.5)	0.188 (1)
	Limb	45 (79.0)	33 (82.5)	
ALS: use of NIV^d^	No	50 (87.7)	34 (85.0)	0.150 (1)
	Yes	7 (12.3)	6 (15.0)	
ALS-informant relationship	Spouse	42 (85.7)	19 (54.3)	***10.142 (1)
	Other	7 (14.3)	16 (45.7)	
Informant identifies as a caregiver	No	3 (6.1)	22 (62.9)	***31.438(1)
	Yes	46 (93.9)	13 (37.1)	
Informant has dependents other than the person with ALS	No	36 (73.5)	28 (80)	0.480 (1)
	Yes	13 (26.5)	7 (20)	
Informant employed	No	28 (57.1)	12 (60)	0.069 (1)
	Yes	21 (42.9)	14 (40)	

	Mean (SD)		*t* (df)
	REM	F2F	
Age of person with ALS (years)	60.6 (9.7)	64.3 (8.6)	2.0 (95)
Age of informant (years)	59.14 (11.3)	59.6 (16.1)	0.2 (82)
Time informant and person with ALS have known each other (years)	31.97 (15.3)	35.6 (16.2)	1.1 (82)
ALS: years of education	16.3 (3.0)	15.7 (4.3)	−0.7 (65)
ALS-FRS-R^e^ score (possible range 0–48)	34.1 (8.1)	34.9 (7.1)	0.5 (94)
ESS^f^ score (possible range 0–24)	4.3 (2.8)	3.8 (2.1)	−0.7 (77)
ALS: IQ estimate^g^ (possible range 42–127)	100.8 (6.6)	106.2 (8.2)	**3.2 (76)h

	Median (IQR)		*U* (z)
	REM	F2F	
Hours per week that informants provide care (if they identify as caregivers)	25.0 (27.0)	28.0 (24.0)	202.5 (1.08)
ALS: time between onset and diagnosis (months)	9.0 (18.0)	13.0 (16.5)	1014.5 (−1.1)
ALS: disease duration (months)	33.0 (15.5)	27.0 (45.0)	1082 (−0.6)

Adapted from Didcote et al. [46]. This is an Open Access article distributed in accordance with the terms of the Creative Commons Attribution (CC BY 4.0) license, which permits others to distribute, remix, adapt and build upon this work, for commercial use, provided the original work is properly cited. See: http://creativecommons.org/licenses/by/4.0/. The table includes formatting changes from the original table and additional material

^a^Remote

^b^Face-to-face

^c^Amyotrophic lateral sclerosis

^d^Non-invasive ventilation

^e^ALS-Functional Rating Scale—Revised

^f^Epworth Sleepiness Scale

^g^IQ estimated using test of premorbid functioning: full-scale IQ

^h^Cohen’s d was above 0.7 for the significant *t*-test

^***^p < 0.001

^**^p < 0.01

Multiple regression models assessed the ability of behavioural screening tools to predict informant distress. See Table 5 in Online Resource 1 for median informant distress scores. The results from the five models (one for each behavioural score) are presented in Table 3 and summarised here.

1. Administration mode was not a significant predictor variable in any of the models, and therefore, it did not affect the predictive relationships between behavioural screening tool scores and informant distress.
2. Informant employment status was a significant covariate in Model 4 which assessed the ability of the MiND-B to predict informant distress but was not a significant predictor variable in any other models.
3. No other significant covariates occurred in any of the final models, including the variable representing whether or not informants identified as a caregiver, so these variables were omitted in step two of the regression analysis.
4. All behavioural screening tools predicted informant distress, in their corresponding separate models (Models 1 to 5). Specifically:
  - For each unit increment in ECASb score, informant distress was higher on average by 0.1 units. Model 1 accounted for 8.3% of the variance in informant distress.
  - Informant distress decreased by 0.1 units, on average, per one unit increment in ALS-CBSb scores (the ALS-CBSb is reverse scored; lower ALS-CBSb scores indicate greater behavioural change). Model 2 accounted for 17.8% variance in informant distress score.
  - Informant distress increased by 0.1 for each unit increase in BBI score. Model 3 accounted for 26.3% of variance in informant distress.
  - For each unit increase in MiND-B, informant distress was expected to be lower on average by 0.1 units (the MiND-B is reverse scored; lower MiND-B scores indicate greater behavioural change), adjusted for employment status. Model 4 accounted for 37.5% of informant distress, the highest of all behavioural screening tool models. Model 4 included the covariates “administration mode” and “informant employment status”, while other models only included “administration mode”. For those informants who were not employed (unemployed, retired, and students), informant distress was 0.3 units lower than for those who were employed. If the regression model only contained “administration mode” and the MiND-B score, it still accounted for the most variance (34.3%) in informant distress (*F*(2, 79) = 20.598, *p* < 0.001, *R*^2^ = 0.343) when considering the three behavioural screening tools.
  - For each unit increment in ALS-FTD-Q score, informant distress increased by 0.03 units. Model 5 accounted for 21.9% of variance in informant distress.

**Table 3:** Multiple regression models to assess whether behavioural screen scores predict informant distress (dependent variable)

Model 1: *F*(2, 79) = 3.596, *p* = 0.032, *R*^2^ = 0.083			
	*B*^a^ (SE^b^)	*t*^c^ (df = 79)	*p*-value
Constant	−0.2 (0.2)	−1.349	0.181
Predictors			
ECASb^d^	0.1 (0.1)	2.437	0.017
Administration mode^e^	0.1 (0.2)	0.587	0.559
Informant employment status	Omitted	Omitted	Omitted

Model 2: *F*(2, 79) = 8.526, *p* < 0.001, *R*^2^ = 0.178			
	*B* (SE)	*t* (df = 79)	*p*-value
Constant	1.8 (0.5)	3.571	0.001
Predictors			
ALS-CBSb^f^	−0.1 (0.01)	−3.956	< 0.001
Administration mode	0.2 (0.2)	0.938	0.351
Informant employment status	Omitted	Omitted	Omitted

Model 3: *F*(2, 76) = 13.570, *p* < 0.001, *R*^2^ = 0.263			
	*B* (SE)	*t* (df = 76)	*p*-value
Constant	−0.5 (0.2)	−2.926	0.005
Predictors			
BBI^g^	0.1 (0.01)	5.151	< 0.001
Administration mode	0.04 (0.2)	0.197	0.844
Informant employment status	Omitted	Omitted	Omitted

Model 4: *F*(3, 76) = 15.194, *p* < 0.001, *R*^2^ = 0.375			
	*B* (SE)	*t* (df = 76)	*p*-value
Constant	4.9 (0.8)	6.375	< 0.001
Predictors			
MiND-B^h^	−0.1 (0.02)	−6.498	< 0.001
Administration mode	0.1 (0.2)	0.641	0.523
Informant employment status	−0.3 (0.2)	−2.137	0.036

Model 5: *F*(2, 79) = 11.070, *p* < 0.001, *R*^2^ = 0.219			
	*B* (SE)	*t* (df = 79)	*p*-value
Constant	−0.6 (0.2)	−3.460	0.001
Predictors			
ALS-FTD-Q^i^	0.03 (0.01)	4.546	< 0.001
Administration mode	0.2 (0.2)	0.902	0.370
Informant employment status	Omitted	Omitted	Omitted

Omitted variables were tested but omitted from the final model, following a forward stepwise procedure

^a^Beta value

^b^Standard error

^c^*t*-value

^d^Behavioural component of the Edinburgh Cognitive and Behavioural ALS Screen; possible scores 0–10 (higher scores indicate greater behavioural change)

^e^Administration mode categories: tested face-to-face/tested remotely

^f^Behavioural component of the ALS Cognitive Behavioural Screen; possible scores 0–45 (higher scores indicate less behavioural change)

^g^Beaumont Behavioural Inventory; possible scores 0–123 (higher scores indicate more behavioural change)

^h^Motor Neuron Disease Behavioural Instrument; possible scores 9–36 (higher scores indicate less behavioural change)

^i^ALS-FTD Questionnaire; possible scores 0–100 (higher scores indicate greater behavioural change)

### Cognitive measures

The ability of cognitive screening tools to predict informant distress was investigated with multiple regression models, one for each cognitive screen. The results are presented in Table 4 and summarised here.

1. Administration mode was not a significant predictor variable in any of the regression models and so did not affect the predictive relationships between cognitive screening tool scores and informant distress.
2. ALSFRS-R score was a significant covariate in the models that assessed the abilities of the ECASc and ALS-CBSc to predict informant distress.
3. Informant age was a significant covariate in the model that assessed the ability of the ECASc to predict informant distress.
4. There were no other significant covariates (including whether or not informants identified as being a caregiver) in any of the final models.
5. The Mini-ACE model did not significantly predict informant distress.
6. Multiple regression models that assessed the ability of the ECASc and ALS-CBSc to predict informant distress were significant (Models 6 and 7).
  - For each unit increase in ECASc score, where a high score indicates better cognitive performance, informant distress decreased on average by 0.03 units. For each unit increase in ALSFRS-R score, where a higher score indicates better physical function, informant distress decreased by 0.03 on average and for each unit increase in age of informant, informant distress decreased by 0.02 on average. Model 6 accounted for 24.9% of informant distress.
  - For each unit increment in ALS-CBSc scores, where a high score indicates better cognitive performance, informant distress decreased on average by 0.1 units and for each unit increment in ALSFRS-R scores, informant distress decreased on average by 0.03 units. Model 7 accounted for 21.8% of informant distress.

**Table 4:** Multiple regression models to assess whether cognitive screen scores predict informant distress (dependent variable)

Model 6: *F*(4, 73) = 5.962, *p* < 0.001, *R*^2^ = 0.249			
	*B*^a^ (SE^b^)	*t*^c^ (df = 73)	*p*-value
Constant	4.6 (1.1)	4.113	< 0.001
Predictors			
ECASc^d^	−0.03 (0.01)	−2.926	0.005
Administration mode^e^	0.3 (0.2)	1.737	0.087
ALSFRS-R^f^	−0.03 (0.01)	−2.175	0.033
Age of informant	−0.02 (0.01)	−2.459	0.016

Model 7: *F*(3, 74) = 6.872, *p* < 0.001, *R*^2^ = 0.218			
	*B* (SE)	*t* (df = 74)	*p*-value
Constant	2.7 (0.7)	4.007	< 0.001
Predictors			
ALS-CBSc^g^	−0.1 (0.03)	−3.396	0.001
Administration mode	0.3 (0.2)	1.145	0.145
ALSFRS-R	−0.03 (0.01)	−2.283	0.025
Age of informant	Omitted	Omitted	Omitted

Model 8: *F*(2, 77) = 0.767, *p* = 0.468, *R*^2^ = 0.020			
	*B* (SE)	*t* (df = 77)	*p*-value
Constant	0.1 (1.2)	0.117	0.907
Predictors			
Mini-ACE^h^	−0.01 (0.2)	1.215	0.813
Administration mode	0.2 (0.2)	−0.237	0.228
ALSFRS-R	Omitted	Omitted	Omitted
Age of informant	Omitted	Omitted	Omitted

Omitted variables were tested but omitted from the final model, following a forward stepwise procedure

^a^Beta value

^b^Standard error

^c^*t*-value

^d^Cognitive component of the Edinburgh Cognitive and Behavioural ALS Screen; possible scores 0–136

^e^Administration mode categories: tested face-to-face/tested remotely

^f^Amyotrophic Lateral Sclerosis Functional Rating Scale—Revised (Higher scores indicate less functional impairment; possible range 0–48)

^g^Cognitive component of the ALS Cognitive Behavioural Screen; possible scores 0–20

^h^Mini-Addenbrooke’s Cognitive Examination; possible scores 0–30

### The interaction between cognitive and behavioural impairment

The behavioural (MiND-B) and cognitive (ECASc) screens that were entered into the model as predictor variables were selected because their relevant regression models had explained the largest percentage of variance in informant distress compared to the other cognitive and behavioural screens assessed here.

The interaction term between the MiND-B and ECASc was entered into the model but it was not a significant predictor of informant distress so was removed from the final model to optimise power. When the MiND-B and ECASc were entered into the model separately, both were significant predictors of informant distress. When the ECASc was introduced to the MiND-B model (this was the final model; see Table 5), the MiND-B remained a significant predictor of informant distress but the ECASc was no longer a significant predictor. This suggests that ALS with behavioural impairment predicts informant distress to a greater extent than ALS with cognitive impairment. Administration mode (whether measures were administered face-to-face or remotely) did not affect the predictive relationships between the screening tool scores and informant distress (see Table 5).

**Table 5:** Multiple regression to assess whether behavioural and cognitive screen scores predict informant distress (dependent variable) within the same model

Model 9: *F*(3, 75) = 14.266, *p* < 0.001, *R*^2^ = 0.365			
	*B*^a^ (SE^b^)	*t*^c^ (df = 75)	*p*-value
Constant	5.5 (1.0)	5.423	< 0.001
Predictors			
MiND-B^d^	−0.1 (0.02)	−5.420	< 0.001
ECASc^e^	−0.01 (0.01)	−1.540	0.128
Administration mode^f^	0.2 (0.2)	1.395	0.167

^a^Beta value

^b^Standard error

^c^*t*-value

^d^Cognitive component of the Edinburgh Cognitive and Behavioural ALS Screen; possible scores 0–136 (high scores indicate less cognitive change)

^e^Motor Neuron Disease Behavioural Instrument; possible scores 9–36 (higher scores indicate less behavioural change)

^f^Administration mode categories: tested face-to-face/tested remotely

## Discussion

Based on the currently administered measures, behavioural impairment in people with ALS predicted informant distress, irrespective of which behavioural screening tool was used. Cognitive impairment in people with ALS predicted informant distress, although only when the ECASc and ALS-CBSc (rather than the Mini-ACE) were used to assess cognitive impairment. The measures of cognitive impairment in ALS and of behavioural impairment in ALS that explained the most variance in informant distress did not interact to predict informant distress. When assessed together, only ALS with behavioural impairment predicted informant distress, not ALS with cognitive impairment.

The range of variance in informant distress, accounted for by the different behavioural screening tools (8.3–34.3%), may be due to differences in the number of items/types of symptoms that most affect informant distress. For example, the ECASb has two items that address hyperorality and food preferences, yet the BBI has six. A larger number of items or symptoms addressing a behavioural domain may be more likely to address the domain from different angles and make the informant more likely to feel that their experience applies to the domain and report it. In addition, when considering the types of symptoms covered, the MiND-B addresses behavioural disinhibition in one of its items by asking whether the informant agrees that “He/she (person with ALS) has temper outbursts”. The ECASb does not assess this symptom. Perhaps temper outbursts cause distress in informants, resulting in the MiND-B accounting for more variance in informant distress than the ECASb.

The results are in line with what has previously been demonstrated with some components of the current measure of informant distress (i.e., burden and anxiety [14, 16–18]). This predictive relationship of behavioural involvement was found here irrespective of which behavioural screening tool was used. Some results, however, conflict with findings reported in the literature. A previous investigation by Siciliano et al. [39] into the ability of the ECASb to predict informant burden, depression, and anxiety did not find a predictive relationship between the ECASb and any of these measures of informant distress. Beyond ECASb scores, Siciliano et al. [39] also included 12 other variables, including the ECASc ALS-specific and non-ALS specific scores; age; education; gender; time dedicated to care; duration of disease; ALS-FRS-R score; Barthel Index (measure of functional dependence) [40]; Coping Inventory for Stressful Situations (CISS; task oriented score, emotion oriented score, and the avoidance oriented score) [41] as predictor variables in the initial regression model. In a second regression analysis, the model still contained the ECASb scores and all of the other variables excluding the CISS scores. In both regression models (conducted separately for the prediction of burden, anxiety, and depression), ECASb scores were not predictive of these measures of informant distress. The differences in findings between Siciliano et al.’s [39] study and the current study may be due to several reasons. First, variables included in the regression models in the current study were fewer than those in the Siciliano et al. [39] study and the variables were different. There may have been an excess of predictor variables in Siciliano et al.’s [39] models, causing issues with overfitting the regressions. Alternatively, the difference in findings may be due to the use of a composite measure in the current study, rather than assessing anxiety, depression and burden independently.

Unlike the ECASb and ALS-CBSb, the Mini-ACE did not significantly predict informant distress (see Table 4). This may be because the Mini-ACE is not specific to ALS and of the three cognitive screening tools, it may not be a good measure of cognitive impairment in people with ALS [42]. The Mini-ACE therefore may be less informative when considering the impact of cognitive impairment in the person with ALS on others.

Fewer studies have focused on cognitive impairment in people with ALS influencing informant distress than have focused on ALS with behavioural impairment. Siciliano et al. [39] found that the ECASc did not predict informant burden, anxiety, and depression. Besides possible issues with overfitting the regression models, another potential reason for the difference in findings is that Siciliano et al. [39] used ECASc ALS-specific and non-ALS-specific scores as predictor variables in regression models, whereas in the current study, ECASc total scores were used.

There are a number of strengths and limitations to this study. This is the first study to investigate whether cognitive and behavioural impairment interact to predict informant distress. The findings demonstrate that cognitive and behavioural changes affect informant distress independently but do not interact in their predictive effect. Therefore, the impact of cognitive and behavioural changes on informant distress should be assessed separately.

Another strength of this research is the distinction made between caregiver and non-caregiver informants, rather than simply referring to recruited informants as ‘caregivers’ without ensuring that they fulfil a caregiving role. A large proportion of non-caregiver informants in samples may lead to underestimation of burden, anxiety, depression and stress, and an overestimation of quality of life. This transparency is, therefore, helpful for those trying to infer the implications of the research and is recommended for future studies. It could be argued that informant distress in the current sample might be underreported because informants might spend time with the person with ALS in a different manner than might caregivers and so might be affected by caregiving to a lesser extent. Nonetheless, it was still possible to determine that cognitive and behavioural impairment in people with ALS are independently predictive of psychosocial and psychological wellbeing in this sample of nominated participants.

In terms of possible limitations, it is possible that the use of a composite measure of informant distress may obscure relationships between the components of the measure and other variables. For instance, demographic variables and cognitive and behavioural screens may have had different predictive relationships to anxiety, depression, or burden compared to the composite measure. Despite this potential limitation, the component variables were moderately to strongly intercorrelated (see Online Resource 1). Thus, by constructing a composite measure, we adopted a conservative approach to the regressions which effectively facilitated the assessment of the overall distress of informants.

Additionally, the sample was mixed in its data collection methods, due to the COVID-19 pandemic and its restrictions. One subsample of people with ALS took part in face-to-face assessments and the other subsample was assessed via videoconferencing with stimuli presented digitally (see Online Resource 1). Similarly, a subsample of the informants completed behavioural ratings and questionnaires on paper and the remainder completed the measures as one cohesive online questionnaire. Although administration mode did not predict informant distress, it is important to acknowledge this inconsistency in data collection.

A further limitation is that the ECAS includes a separate psychosis screening component that was not part of this analysis. The psychosis screen was omitted in the current study as it is not formally part of the ECASb, does not influence the behavioural screen score, and is not mentioned in the interpretation guidelines for the behavioural screen [43]. In contrast, other behavioural screening tools lack a dedicated psychosis screen, although the BBI and ALS-FTD-Q do feature two items related to psychosis. Had the psychosis screen been considered in behavioural impairment score of the ECAS, a stronger relationship between ECASb and informant distress may have been found.

Furthermore, we were unable to undertake a detailed comparison of distress levels across different informant types (e.g., spouses, family members, and friends) as statistical power was insufficient. Future research with larger, more diverse samples of informants could explore whether relationships between cognitive and behavioural impairment in the person with ALS and caregiver distress differ based on the informant’s relationship to the patient.

For 52.5% of ALS participants in the face-to-face condition and 33.3% in the remote condition, muscle wasting in the arms and hands prevented them completing one item in either the ALS-CBSc (raising arms and making a fist) and/or one item in the Mini-ACE (drawing a clock). Where this was the case, participants were awarded full marks for the task (see Online Resource 1 for further information). It is possible, therefore, that these participants’ ALS-CBSc and Mini-ACE scores underestimated their level of cognitive impairment which may have influenced whether these cognitive screens were predictive of informant distress. The ECASc, which was tested in the final regression models, was unaffected by physical symptoms of ALS in this sample.

It was not possible to establish consistent formal diagnostic classifications of ALS with cognitive impairment, ALS with behavioural impairment, ALS with cognitive and behavioural impairment or ALS-FTD for the sample within the scope of the study, given the range of measures used and their different cut-off scores. It is possible that those people with ALS who had greater evidence of cognitive impairment also had behavioural impairment or even ALS-FTD but it would seem that the level of behavioural change is what particularly influences informant distress. Future research should assess the impact of cognitive impairment, behavioural impairment, whether they are comorbid or occur in the context of full-blown ALS-FTD when considering their role in predicting informant distress.

There is some evidence that depression in people with ALS is linked to depression or poor mental health in caregivers [44, 45]. Since depression was not measured in the current sample of people with ALS, future research should assess the relationships between cognitive and/or behavioural involvement in people with ALS and informant distress while controlling for depression in people with ALS to rule out this potential confounding variable.

In summary, this study has demonstrated that behavioural impairment in people with ALS predicts informant distress, irrespective of which of the specific behavioural screening tool is used here. The variability in the percentage of informant distress explained by ALS behavioural impairment using different screens suggests that the number and scope of items addressing behavioural symptoms can significantly affect the outcomes. Although cognitive impairment also contributes to informant distress, its impact is less pronounced, and its ability to predict informant distress varies depending on the specific tool employed. Notably, the interaction between cognitive and behavioural impairments did not significantly predict informant distress. Future research should address limitations such as the exclusion of the ECAS psychosis screen and the need for more detailed reporting on informant–caregiver relationships, to better understand and mitigate the impact of ALS on informants’ wellbeing.

## Supplementary Information

Below is the link to the electronic supplementary material.

Supplementary file1 (DOCX 176 KB)

## Data Availability

Anonymised data may be available on reasonable request.
