# Supplementary material for: Predicting ALS informant distress from cognitive and behavioural change in people with ALS
Source: J Neurol. 2025 Jan 15;272(2):144. doi: 10.1007/s00415-024-12847-7 (PMC11735582; doi:10.1007/s00415-024-12847-7)
Supplement: Supplementary file 1 — Supplementary file1 (DOCX 176 KB) [file 415_2024_12847_MOESM1_ESM.docx]

# Online Resource 1

Title: Predicting ALS informant distress from cognitive and behavioural change in people with ALS

Journal: Journal of Neurology

Lyndsay Didcote^a^, Silia Vitoratou^b^, Ammar Al-Chalabi*^c,d^ & Laura H. Goldstein^a^

a = Department of Psychology, Institute of Psychiatry, Psychology and Neuroscience, King’s College London, London, UK;

b =Department of Biostatistics and Health Informatics, Institute of Psychiatry, Psychology and Neuroscience, King’s College London, London, UK;

c = Department of Basic and Clinical Neuroscience, Maurice Wohl Clinical Neuroscience Institute, King’s College London, London, UK;

d = Department of Neurology, King’s College Hospital NHS Foundation Trust, London, UK;

*Corresponding author Professor Ammar Al-Chalabi, K0.27. Maurice Wohl Clinical Neuroscience Institute, 5 Cutcombe Road, London SE5 9RX. Email [ammar.al-chalabi@kcl.ac.uk](about:blank)

Caption describing content of this file: This file contains details of cognitive measures behavioural measures, measures of informant distress, additional detail on data collection for the cognitive screening tests, a table demonstrating correlations between measures of informant distress, median distress scores for informants and figures showing recruitment pathways.

## Cognitive screening tools

Table 1: Descriptions of cognitive screening tools.

| ECASc^a^ [1] | ALS^d^-specific screening tool that takes around 15 minutes to administer. It evaluates executive function (including social cognition), verbal fluency, and language. It also assesses the memory and visuospatial function, which are not ALS-specific. PwALS^e^ can respond orally or in writing for all items.  ALSci^f^ cut-off score: ≤ 105/136 and the ALS Total score was used here. |
| --- | --- |
| ALS-CBSc^b^ [2] | ALS-specific screening tool that takes around 5 minutes to administer. It evaluates concentration; verbal fluency; attention; mental tracking and monitoring. The command task for the attention assessment is not possible for some pwALS with arm and hand muscle wasting that prevents dexterity (see Didcote et al., [3]).  ALSci cut-off score: < 17/20  ALS-FTD^g^ cut-off score: ≤ 10/20. The total ALS-CBSc score was used here. |
| Mini-ACE^c^ [4] | This screening tool is not ALS-specific and takes around 5 minutes to administer. It assesses memory, fluency, attention, and visuospatial function. The clock drawing task cannot be completed for those with arm and hand muscle wasting that prevents dexterity and writing (see Didcote et al., [3]).  ALS-FTD cut-off score: ≤ 25/30. The total Mini-ACE score was used here. |

^a^ Cognitive component of the Edinburgh Cognitive and Behavioural ALS Screen. ^b^ Cognitive component of the ALS Cognitive Behavioral Screen. ^c^ Mini-Addenbrooke’s Cognitive Examination. ^d^ Amyotrophic lateral sclerosis. ^e^ People with ALS. ^f^ ALS with cognitive impairment. ^g^ ALS-frontotemporal spectrum disorder. Table adapted from Didcote et al. [3]. This is an Open Access article distributed in accordance with the terms of the Creative Commons Attribution (CC BY 4.0) license, which permits others to distribute, remix, adapt and build upon this work, for commercial use, provided the original work is properly cited. See: http://creativecommons.org/licenses/by/4.0/. The table includes wording and formatting changes from the original table.

## Additional detail on data collection for cognitive screening tests

To facilitate responses via tablet, laptop, or desktop PC in the remote condition, modifications were made to the response format for cognitive screens. While participants could point to select stimuli in person, they were required to either name or describe their choice or indicate the location of the selected item among others in the remote setting. For the verbal fluency tasks conducted remotely, participants wrote their answers on paper as they would in person, then displayed the paper to the webcam for scoring. For tasks involving alternating numbers and letters in the ECASc and ALS-CBSc, participants wrote each set (a number and a letter) on separate sheets of paper, flipping the page to continue with the next set. In the face-to-face condition, responses that were written due to bulbar symptoms were entered into the chat function of the videoconferencing software.

Some participants were unable to perform certain tasks due to their physical limitations. For instance, participants with muscle wasting in their arms and hands could not complete certain actions in the ALS-CBSc, such as raising their arms or making a fist, and were also unable to perform the clock-drawing task in the Mini-ACE. In these instances, participants were awarded full credit for the tasks they could not physically complete.

It was not deemed feasible to assign zero marks to participants unable to complete cognitive screening test items d due to motor impairment where no alternative completion mode was possible because in other aspects of our group’s work total scores were utilized to gauge impairment via cut-off scores, and this approach could have artificially lowered scores to the point where participants might be incorrectly classified as impaired. Factor analysis has not proven that the ALS-CBCc and Mini-ACE are unidimensional; these tests evaluate multiple cognitive domains, typically with only a single task per domain. Consequently, pro-rating scores was deemed unsuitable. In addition, omitting participants who could not complete certain tasks would have compromised the statistical power for analyses and reduced the ability to generalise findings to the broader ALS population.

Table 2: Descriptions of behavioural screening tools.

| ECASb^a^ [1] | This screening tool contains 10 items and takes 15 minutes to administer. It is originally administered as an interview and, based on the Rascovsky et al., [5] criteria, detects behavioural-variant FTD. ALS^f^ behavioural domains assessed are: loss of sympathy or empathy; behavioural disinhibition; perseverative, stereotyped, compulsive or ritualistic behaviour; hyperorality and altered food preference; apathy or inertia.  ALSbi classification: score of at least 1 in the apathy domain OR score of at least 1 in 2/5 domains.  ALS-FTD^g^ classification: score of at least 1 in 3/5 domains. The total number of behaviours identified was used here for the analyses. |
| --- | --- |
| ALS-CBSb^b^ [2] | This 19-item questionnaire takes around 5 minutes to administer. It evaluates decision making; language; judgement; apathy; emotional control; insight; inhibition; frustration tolerance; empathy; food preferences; and cognitive flexibility.  ALSbi^h^ cut-off score: ≤ 36/45  ALS-FTD cut-off score: ≤ 32/45. The total ALS-CBSb score was used here. |
| BBI^c^ [6] | This 41-item questionnaire screening tool takes around 20 minutes to administer. It evaluates altered response to sensory stimuli; dietary changes; social cognition; cognitive change (executive function and language); behavioural disinhibition; behavioural stereotypes; egocentricity; echolalia; apathy; obsessive-compulsive behaviours; utilisation behaviour; and the psychosis symptoms of hallucinations and delusions.  ALSbi cut-off score: ≥ 7/123  ALS-FTD cut-off score: ≥ 22.5 /123. The total BBI score was used here. |
| MiND-B^d^ [7] | This questionnaire screening tool contains 9-items and takes around 5 minutes to administer. It assesses apathy, stereotypical behaviour, and disinhibition.  ALSbi cut-off score: ≤ 35/36. The total MiND-B score was used here. |
| ALS-FTD-Q^e^ [8] | This questionnaire screening tool takes around 10 minutes to administer and contains 25 items. Domains evaluated include disinhibition; egocentricity; apathy; emotional lability; irritability; altered food preference; hallucinations; delusions (paranoia); and cognition (memory, orientation, and cognition).  ALSbi cut-off score: ≥ 22/100  ALS-FTD cut-off score: ≥ 29/100. The total ALS-FTD score was used here. |

^a^ Behavioural component of the Edinburgh Cognitive and Behavioural ALS Screen​. ^b^ Behavioural component of the ALS Cognitive Behavioral Screen. ^c^ Beaumont Behavioural Inventory. ^d^ Motor Neuron Disease Behavioural Instrument. ^e^ ALS-FTD Questionnaire. ^f^ Amyotrophic lateral sclerosis. ^g^ Frontotemporal dementia. ^h^ ALS behavioural impairment. Adapted from Didcote et al [9]. This is an Open Access article distributed in accordance with the terms of the Creative Commons Attribution (CC BY 4.0) license, which permits others to distribute, remix, adapt and build upon this work, for commercial use, provided the original work is properly cited. See: http://creativecommons.org/licenses/by/4.0/. The table includes wording and formatting changes from the original table.

| Table 3: Descriptions of measures of psychological and psychosocial distress in informants. | |
| --- | --- |
| Screening tool | Description |
| PHQ-9^a^[10] | The 9-item depression module from the PHQ. It assesses depressed mood, anhedonia, poor appetite or overeating, sleeping too much or too little, slowing of speech or movement noticed by others (or fidgeting and restlessness), loss of energy or feeling tired, feelings of worthlessness or guilt, diminished ability to concentrate, recurrent thoughts of death. Each item is scored from 0-3 with higher scores indicating greater depression. Possible range of scores is 0-27; the cut-off score indicating moderate depression is 10 or above. The PHQ-9 has good internal consistency, test-retest reliability, test validity, and construct validity [10–12]. |
| GAD-7^b^[13] | The GAD-7 assesses difficulty relaxing, irritability, fear of something awful happening, restlessness, worrying too much about different things, not being able to stop or control feelings of worry, and feeling nervous/anxious. Each of the seven items is scored from 0-3 with higher scores indicating greater anxiety. Possible range of scores is 0-21; the cut-off score indicating generalised anxiety disorder is 10 or above. The GAD-7 has good internal consistency, test validity, and construct validity [13–15]. |
| ZBI^c^[16] | This 12-item measure of caregiver burden assesses anger towards the person for whom they are a caregiver, feeling as though the caregiver does not have enough time for themselves, feeling stressed because of balancing caregiving and other responsibilities, feeling the person for whom they care affects the relationships that the caregiver has with others, feelings of strain when around the person for whom they are a caregiver, poorer health as a result of being a caregiver, reduced privacy as a result of being a caregiver, poorer social life as a result of being a caregiver, the caregiver feeling as though they have lost control of their life since they became a caregiver, feelings of uncertainty about what to do about the person for whom they are a caregiver, the caregiver feeling as though they should be doing more for the person for whom they care, and the caregiver feeling as though they could do a better job of caregiving. Items are scored 0-4 with higher scores indicating higher caregiver burden. Possible range of scores is 0-48; the cut-off score indicating caregiver burden is 17 or above. The ZBI has good internal consistency, test validity and construct validity [16–18]. |

^a^ Patient Health Questionnaire. ^b^ Generalized Anxiety Disorder Assessment. ^c^ Zarit Burden Interview – short form.

## Correlations between measures of informant distress

Table 4: Spearman’s correlations between scores on the ZBI, GAD-7, and PHQ-9

|  |  | ZBI^c^  *rho* (n) | PHQ-9  *rho* (n) |
| --- | --- | --- | --- |
| PHQ-9^a^ |  |  |  |
|  | F2F^d^ | 0.596* (n =35) |  |
|  | REM^e^ | 0.626* (n = 48) |  |
| GAD-7^b^ |  |  |  |
|  | F2F | 0.572* (n = 34) | 0.693* (n = 34) |
|  | REM | 0.623* (n = 48) | 0.749* (n = 48) |

*= *p* < 0.001. ^a^ Patient Health Questionnaire [10]. ^b^Generalized Anxiety Disorder Assessment [13]. ^c^ Zarit Burden Interview [16]. ^d^ Face-to-face. ^e^ Remote.

## Median informant distress scores

Table 5: Median PHQ-9, GAD-7, and ZBI scores for informants

|  | | Median (IQR) |
| --- | --- | --- |
| PHQ-9^a^ | |  |
|  | F2F^d^ (n = 35) | 5 (5.0) |
|  | REM^e^ (n = 48) | 5 (6.8) |
| GAD-7^b^ | |  |
|  | F2F (n = 34) | 3.5 (5.5) |
|  | REM (n = 48) | 4.5 (8.5) |
| ZBI^c^ | |  |
|  | F2F (n = 35) | 10.0 (11.0) |
|  | REM (n = 48) | 13.5 (17.0) |

^a^ Patient Health Questionnaire [10]. ^b^ Generalized Anxiety Disorder Assessment [13]. ^c^ Zarit Burden Interview – short version [16]. ^d^ Face-to-face. ^e^ Remote.

## Recruitment pathway figures

Figures were created in Microsoft Word version 2312.

**Fig1**: ALS participant recruitment pathway.

A Flowchart describing the recruitment pathway for the ALS participants. The numbers of participants not included in the final sample due to loss of contact, deciding not to participate, meeting exclusion criteria, who gave consent, who were included in analysis are given

ALS^a^ sample

Identified or volunteered during recruitment phase (F2F^b^ = 69; REM^c^ = 109)

Contact lost prior to giving consent (F2F = 24; REM = 20)

Chose not to participate prior to giving consent (REM = 2)

Screened against exclusion criteria (F2F = 45; REM = 87)

Excluded (F2F = 4; REM = 27)

- Scored above 10 on Epworth Sleepiness Scale

(F2F = 4; REM = 3)

- Not based in UK (REM = 2)
- Over age 75 (REM = 6)
- Diagnosis of PLS^d^

(REM = 10)

- Anarthric and unable to write or type (REM = 6)

Consented

(F2F = 41; REM = 60)

Contact lost after consent was given (REM = 3)

Included in analysis

(F2F = 41; REM = 57)

^a^ Amyotrophic lateral Sclerosis. ^b^ Face-to-face. ^c^ Remote. ^d^ Primary lateral sclerosis. Adapted from Didcote et al [9]. This is an Open Access article distributed in accordance with the terms of the Creative Commons Attribution (CC BY 4.0) license, which permits others to distribute, remix, adapt and build upon this work, for commercial use, provided the original work is properly cited. See: http://creativecommons.org/licenses/by/4.0/. The figure combines data from two previous figures.

**Fig2**: Informant participant recruitment pathway.

A Flowchart describing the recruitment pathway for the informant participants. The numbers of participants not included in the final sample due to loss of contact, deciding not to participate, meeting exclusion criteria, who gave consent, who were included in analysis are given

Decided not to participate after giving consent (REM = 1)

Consented

(F2F = 35; REM = 50)

Contact lost prior to giving consent (F2F = 12; REM = 3)

Informant sample

Nominated by ALS^a^ participants during recruitment phase (F2F^b^ = 50; REM^c^ = 60)

Nominated by ALS participants but did not respond to communication (F2F = 3; REM = 7)

Included in analysis

(F2F = 35; REM = 49)

^a^ Amyotrophic lateral Sclerosis. ^b^ Face-to-face. ^c^ Remote. Adapted from Didcote et al [9]. This is an Open Access article distributed in accordance with the terms of the Creative Commons Attribution (CC BY 4.0) license, which permits others to distribute, remix, adapt and build upon this work, for commercial use, provided the original work is properly cited. See: http://creativecommons.org/licenses/by/4.0/. The figure combines data from two previous figures.

## References

1. Abrahams S, Newton J, Niven E, Foley J, Bak TH (2014) Screening for cognition and behaviour changes in ALS. Amyotroph Lateral Scler Frontotemporal Degener 15:9–14

2. Woolley SC, York MK, Moore DH, Strutt AM, Murphy J, Schulz PE, Katz JS (2010) Detecting frontotemporal dysfunction in ALS: Utility of the ALS Cognitive Behavioral Screen (ALS-CBS^TM^). Amyotrophic Lateral Sclerosis 11:303–311

3. Didcote L, Vitoratou S, Al-Chalabi A, Goldstein LH (2024) Comparison of in-person vs. remote administration of cognitive screening tools for people with ALS. Neurological Sciences 1–9

4. Hsieh S, McGrory S, Leslie F, Dawson K, Ahmed S, Butler CR, Rowe JB, Mioshi E, Hodges JR (2015) The Mini-Addenbrooke’s Cognitive Examination: A new assessment tool for dementia. Dement Geriatr Cogn Disord 39:1–11

5. Rascovsky K, Hodges JR, Knopman D, et al (2011) Sensitivity of revised diagnostic criteria for the behavioural variant of frontotemporal dementia. Brain 134:2456–2477

6. Elamin M, Pinto-Grau M, Burke T, et al (2017) Identifying behavioural changes in ALS: Validation of the Beaumont Behavioural Inventory (BBI). Amyotroph Lateral Scler Frontotemporal Degener 18:68–73

7. Mioshi E, Hsieh S, Caga J, et al (2014) A novel tool to detect behavioural symptoms in ALS. Amyotroph Lateral Scler Frontotemporal Degener 15:298–304

8. Raaphorst J, Beeldman E, Schmand B, et al (2012) The ALS-FTD-Q: A new screening tool for behavioral disturbances in ALS. Neurology 79:1377–1383

9. Didcote L, Vitoratou S, Al-Chalabi A, Goldstein LH (2024) The reliability and validity of in-person and remote behavioural screening tools for people with amyotrophic lateral sclerosis. J Neurol Sci. https://doi.org/10.1016/j.jns.2024.123282

10. Kroenke K, Spitzer RL, Williams JBW (2001) The PHQ-9 validity of a brief depression severity measure. J Gen Intern Med 16:606–613

11. Levis B, Benedetti A, Thombs BD (2019) Accuracy of Patient Health Questionnaire-9 (PHQ-9) for screening to detect major depression: individual participant data meta-analysis. BMJ. https://doi.org/10.1136/bmj.l1476

12. Sun Y, Fu Z, Bo Q, Mao Z, Ma X, Wang C (2020) The reliability and validity of PHQ-9 in patients with major depressive disorder in psychiatric hospital. BMC Psychiatry. https://doi.org/10.1186/s12888-020-02885-6

13. Spitzer RL, Kroenke K, Williams JBW, Löwe B (2006) A brief measure for assessing generalized anxiety disorder. Arch Intern Med 166:1092–1097

14. Löwe B, Decker O, Müller S, Brähler E, Schellberg D, Herzog W, Yorck Herzberg P (2008) Validation and standardization of the Generalized Anxiety Disorder screener (GAD-7) in the general population. Med Care 46:266–274

15. Ruiz MA, Zamorano E, García-Campayo J, Pardo A, Freire O, Rejas J (2011) Validity of the GAD-7 scale as an outcome measure of disability in patients with generalized anxiety disorders in primary care. J Affect Disord 128:277–286

16. Bédard M, Molloy DW, Squire L, Dubois S, Lever JA (2001) The zarit burden interview: a new short version and screening version. Gerontologist 41:652–657

17. Seng BK, Luo N, Ng WY, Lim J, Chionh HL, Goh J, Yap P (2010) Validity and reliability of the Zarit Burden Interview in assessing caregiving burden. Annals Academy of Medicine 39:758–763

18. Yu J, Yap P, Ming Liew T (2019) The optimal short version of the Zarit Burden Interview for dementia caregivers: diagnostic utility and externally validated cutoffs. Aging Ment Health 23:708–710
